# Hydrogen sulfide negatively regulates cd-induced cell death in cucumber (*Cucumis sativus* L) root tip cells

**DOI:** 10.1186/s12870-020-02687-8

**Published:** 2020-10-21

**Authors:** Shilei Luo, Zhongqi Tang, Jihua Yu, Weibiao Liao, Jianming Xie, Jian Lv, Zhi Feng, Mohammed Mujitaba Dawuda

**Affiliations:** 1 College of Horticulture, Gansu Agricultural University, Lanzhou, 730070 China; 2 Horticulture Department, FoA University For Development Studies, Box TL, 1350 Tamale, Ghana

**Keywords:** Mitochondria, Cyt c, Oxidative damage, Cell death, Caspase-3-like protease

## Abstract

**Background:**

Hydrogen sulfide (H_2_S) is a gas signal molecule involved in regulating plants tolerance to heavy metals stress. In this study, we investigated the role of H_2_S in cadmium-(Cd-) induced cell death of root tips of cucumber seedlings.

**Results:**

The results showed that the application of 200 μM Cd caused cell death, increased the content of reactive oxygen species (ROS), chromatin condensation, the release of Cytochrome c (Cyt c) from mitochondria and activated caspase-3-like protease. Pretreatment of seedlings with 100 μM sodium hydrogen sulfide (NaHS, a H_2_S donor) effectively alleviated the growth inhibition and reduced cell death of root tips caused by Cd stress. Additionally, NaHS + Cd treatment could decrease the ROS level and enhanced antioxidant enzyme activity. Pretreatment with NaHS also inhibited the release of Cyt c from the mitochondria, the opening of the mitochondrial permeability transition pore (MPTP), and the activity of caspase-3-like protease in the root tips of cucumber seedling under Cd stress.

**Conclusion:**

H_2_S inhibited Cd-induced cell death in cucumber root tips by reducing ROS accumulation, activating the antioxidant system, inhibiting mitochondrial Cyt c release and reducing the opening of the MPTP. The results suggest that H_2_S is a negative regulator of Cd-induced cell death in the root tips of cucumber seedling.

## Background

Cadmium (Cd) pollution of the environment as a result of human activities has attracted worldwide attention [1]. Cd toxicity can cause ROS elevation in plants, oxidative damage, lipid peroxidation, cell death and growth inhibition [2–4]. Plants under Cd stress exhibit symptoms such as leaf curling and chlorosis [5]. The elongation of roots and synthesis of photosynthetic pigments in wheat was inhibited under Cd stress [6, 7]. Moreover, photosynthesis, synthesis of amino acids and proteins as well as plant growth were all decreased in spinach plants under Cd stress [8]. Plants have developed physiological and biochemical mechanisms to cope with complex and harsh environments and one of such self-defense mechanisms are the accumulation of hydrogen sulfide (H_2_S).

Hydrogen sulfide (H_2_S), which is a vital part of reactive sulfur species [9], has recently been named as the third gasotransmitter, after nitric oxide (NO) and carbon monoxide (CO) [10]. In humans, H_2_S is involved in blood flow, neurotransmission, immune response, hormone secretion and muscle contraction systems [11, 12]. In plants, low concentrations of H_2_S have resulted in characteristics of a gas signal molecule and it has been shown that plants can synthesize endogenous H_2_S under biotic and abiotic stress conditions [13–16]. H_2_S can be produced by D-cysteine desulfhydrase and β-cyano-alanine synthase [17]. H_2_S is involved in regulating plant growth and development, such as inducing adventitious roots formation and regulating stomatal closure [18, 19]. It has also been linked to plants response to environmental stimuli, such as salt, heavy metals (HMs), drought, heat and cold stresses, as well as pathogen infections, which may improve the stress tolerance in plants [20–23].

Programmed cell death (PCD) is a process activated and actuated by the cell itself and it is a well-organized phenomenon at the genetic and biochemical levels. PCD is an important process by which plants respond to environmental changes. PCD involves several processes, including growth and development, as well as plants adaptations to various adverse environmental conditions [24]. Many studies have indicated that PCD can limit development and reproduction, and is also involved in senescence [25, 26] and other processes such as growth [27, 28] and abiotic stress [29]. A high concentration of Cd can induce PCD or necrosis in tomato, tobacco, and *Arabidopsis* cells [30, 31]. Mitochondria play a key role in cellular metabolism and they are key players in the regulation of PCD. Mitochondria are participants in ROS-mediated PCD events, whereas mitochondrial transmembrane potential (MTP) is also reported to be essential in PCD [32]. The mitochondria play an important role in the process of ROS-mediated PCD [33]. Mitochondrial-mediated PCD in animal cells activates caspase protease by releasing apoptotic protease activating factor (Apaf-1) and Cyt c because of the opening of the MPTP [34]. Cyt c release from mitochondrial has been reported in numerous in vitro stress models of plant PCD [35, 36]. Many studies have shown that there is a similar phenomenon in plants [37]. Additionally, the release of Cyt c and activation of caspase-3-like protease were also observed in the process of heat stress-induced PCD in tobacco cells [38], but no studies have shown that the hydrolysis cascade of a single protein in any plant is related to the PCD-related process [39]. These studies suggest that there may be cell death mechanisms similar to that of animal cell apoptosis in plants.

At present, several research studies have been conducted on the stress alleviation role of H_2_S but little research has been conducted on the role of H_2_S in the cell death of plants. Moreover, the effects of H_2_S on cell death in plant are also unclear. The aim of this study was to explore the role of H_2_S in the signaling event participating in cell death in cucumber seedlings under Cd stress.

## Results

### Root length and fresh weight of cucumber seedlings under cd stress

**Fig. 1:**
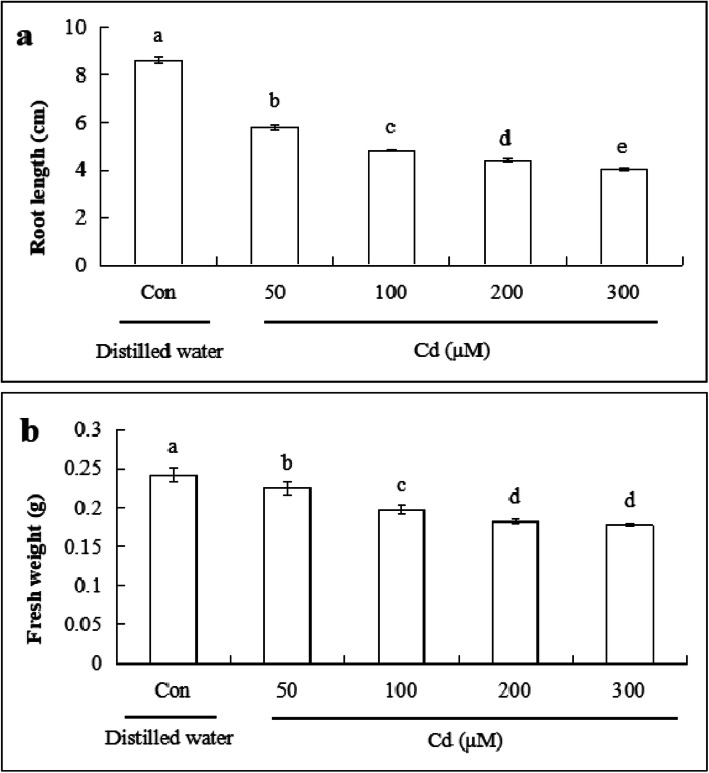
Effects of Cd stress on root length and fresh weight of cucumber seedlings. **a** Root length under Cd stress. **b** Fresh weight of seedlings under Cd stress. Seeds germinated for 2 d were exposed to different concentrations of CdCl_2_ (50, 100, 200, and 300 μM) for 48 h. The data are means ± SE of three independent experiments (*n* = 15). Different letters indicate significant differences (*P* < 0.05; Duncan’s multiple range test)

To investigate the effect of Cd on root length and fresh weight of cucumber seedlings, different concentrations of Cd were applied for 48 h. As shown in Fig. 1, with the increase in Cd concentration, root length and fresh weight decreased significantly. Compared with that of the control, 50 μM Cd caused 33.0, and 7.6% reduction in root length and fresh weight, respectively. The 100 μM Cd dose resulted in a reduction of 44.0% in root length and 20.5% in fresh weight. The 200 μM dose decreased root length by 49.2% and fresh weight by 27.1%. Cd at 300 μM caused 53.1, and 28.9% in root length and fresh weight, respectively. When the concentration was 200 μM, root length was approximately half that of control. Thus, 200 μM CdCl_2_ was chosen for further experiments.

### Cell death of root tips under cd stress

**Fig. 2:**
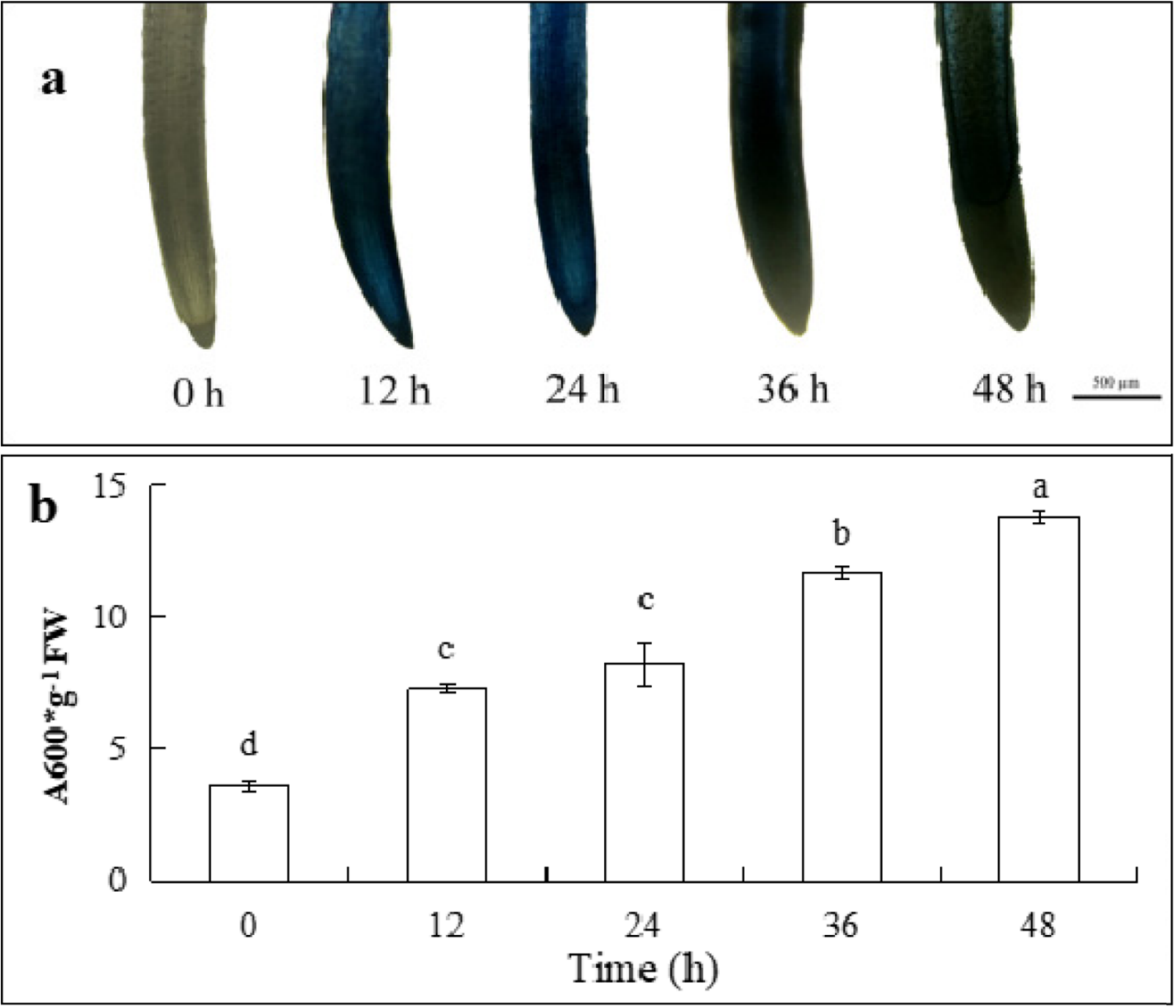
Effects of 200 μM Cd treatment on cell death in root tips of cucumber seedlings. **a** Roots stained with Evans blue at different times (0, 12, 24, 36, and 48 h). **b** Quantitative analysis of root tip cell death in cucumber seedlings. Scale bar indicates 500 μm. The data are means ± SE of three independent experiments. Different letters indicate significant differences (*P* < 0.05; Duncan’s multiple range test). FW, fresh weight

Because Evans blue can stain dead cells, it is indicative of the level of dead cells. As shown in Fig. 2a, with increase in treatment duration (0, 12, 24, 36 and 48 h), root tip staining deepened gradually, indicating that the number of dead root tip cells increased gradually under the influence of Cd. The content of Evans blue in root tips treated for 12 h was significantly higher than that of control (Fig. 2b), and after 48 h of Cd treatment, the content was 2.8 times higher than that of control. These results indicated that Cd inhibited root elongation by causing the death of root cells.

### Effect of H_2_S on root growth and cell death in root tips

**Fig. 3:**
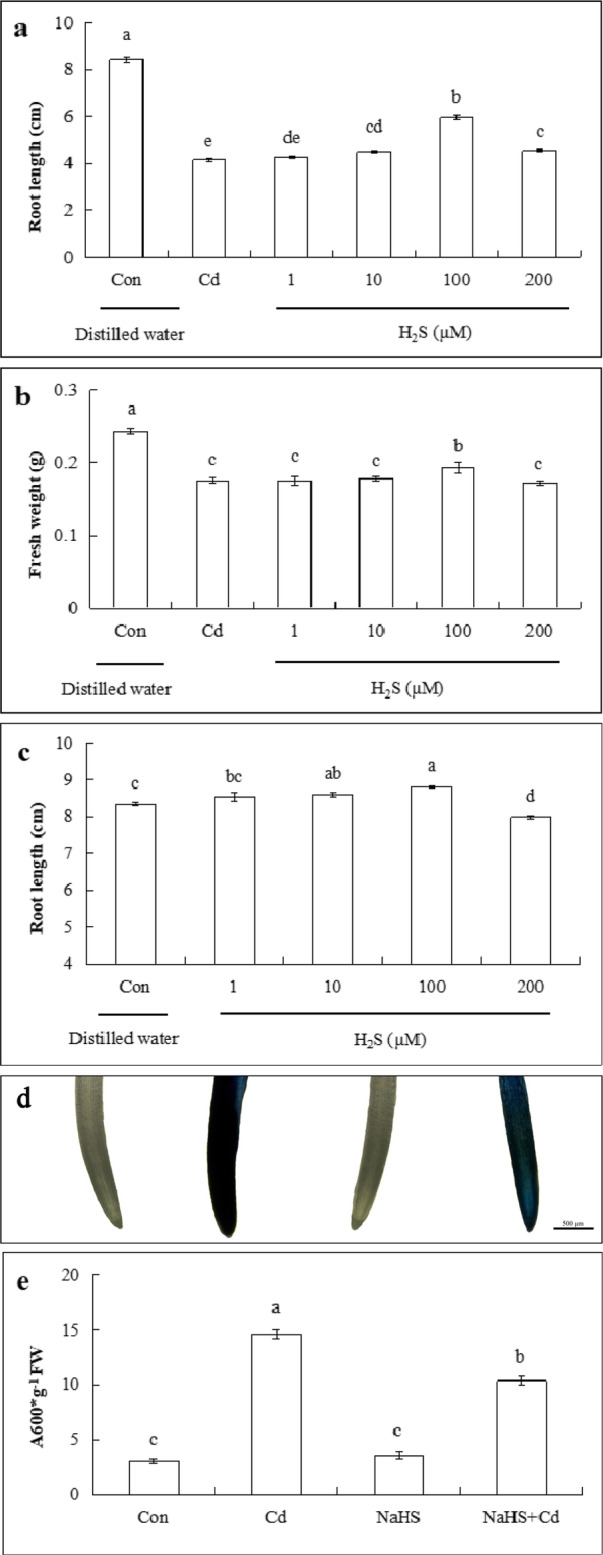
Effects of NaHS on root length, fresh weight and cell death of cucumber seedlings under Cd stress. **a** Effects of different concentrations (1, 10, 100 and 200 μM) of NaHS (a H_2_S donor) on root length (**b**) and fresh weight of cucumber seedlings under Cd stress. **c** Effects of different concentrations (1, 10, 100 and 200 μM) of NaHS on root length of cucumber seedlings under without Cd stress. **d** Roots stained with Evans blue were observed. **e** Quantitative analysis of root tip cell death in cucumber seedlings. Scale bar indicates 500 μm. The results are means ± SE of three independent experiments (*n* = 10). Different letters indicate significant differences (*P* < 0.05; Duncan’s multiple range test). FW, fresh weight

To select the appropriate concentration of NaHS to alleviate Cd stress, the effects of NaHS pretreatment with different concentrations (1, 10, 100 or 200 μM) on root length and fresh weight of cucumber seedlings under Cd stress were observed. As shown in Fig. 3a and b, as the NaHS concentration increased, cucumber root length and fresh weight increased at first and then decreased. Compared with the 200 μM Cd treatment, the 1 μM NaHS caused a 2.7 and 0.1% increase in root length and fresh weight of seedlings, respectively. The 10 μM NaHS treatment increased root length and fresh weight by 8.1, and 1.5% compared with that of Cd alone respectively. Both indices reached the highest values when pretreated with 100 μM NaHS under Cd stress, which resulted in 42.9 and 10.3% greater root length and fresh weight, respectively, than that of Cd treatment. However, seedlings treated with the highest concentration of NaHS (200 μM) exhibited a decrease in effects compared with that of 100 μM NaHS treatment. Figure 3c showed that in the absence of Cd stress, the concentration of NaHS between 1 and 100 μM could promote root length, whereas root length at 200 μM NaHS treatment was significantly lower than those of the other concentrations (1, 10 and 100 μM). These results indicated that the appropriate concentration of NaHS (100 μM) could promote root elongation of cucumber seedlings under Cd stress. Therefore, the 100 μM NaHS was used in the following experiment.

As shown in Fig. 3d, Evans blue staining was observed under different treatments. Cd stress exhibited a deeper staining, whereas the control, the NaHS alone, and the NaHS + Cd treatment exhibited a lighter staining. Figure 3e illustrates that cell death caused by Cd stress was 3.77 times more than the control, whereas NaHS pretreatment reduced cell death by 29.2% compared with Cd treatment alone. There was no difference between values for the control and that of the NaHS treatment alone.

### Endogenous H_2_S in cucumber seedling roots

**Fig. 4:**
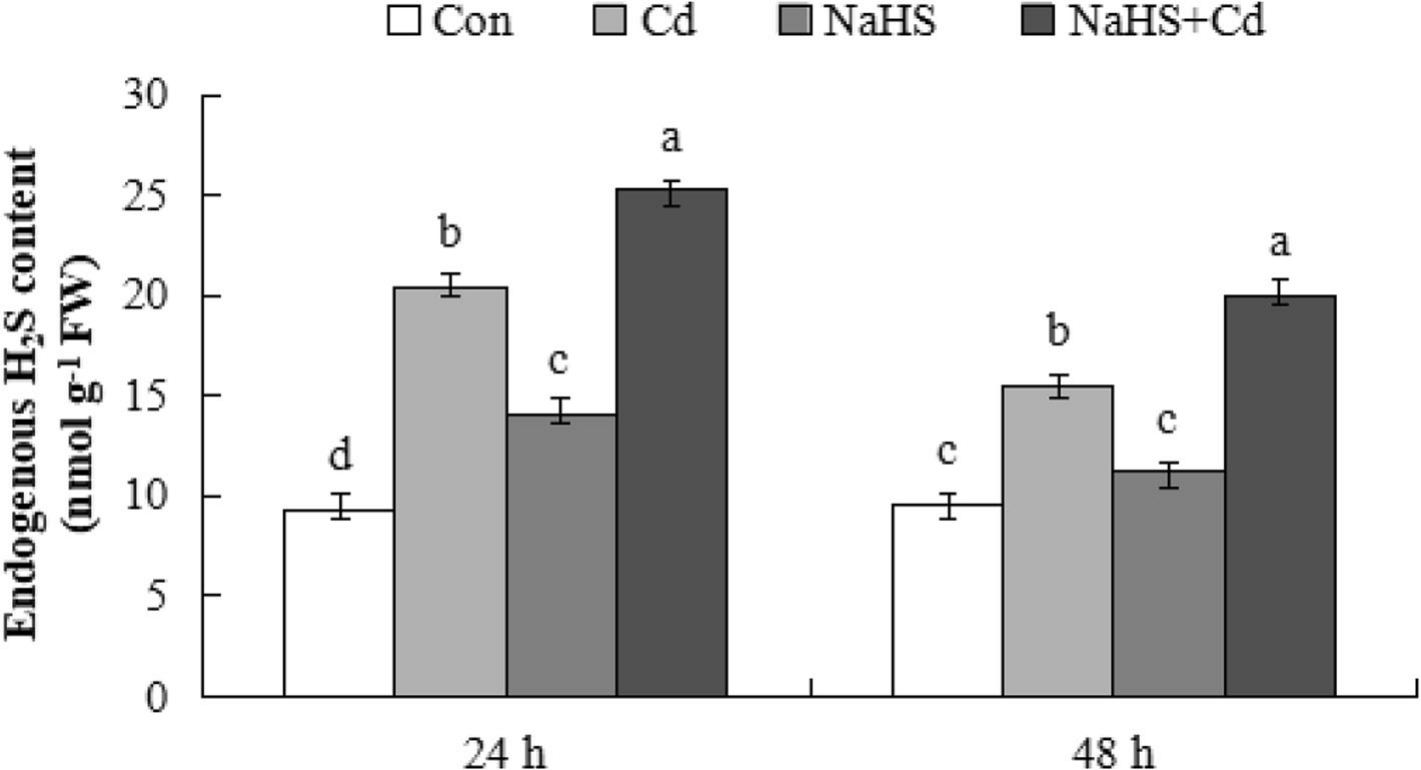
Effects of different treatments on endogenous H_2_S content in cucumber seedling roots. Cucumber seedlings treated with distilled water (Con), 200 μM CdCl_2_ for 48 h, 100 μM NaHS pretreatment for 24 h or 100 μM NaHS pretreatment + Cd for 48 h. The content of endogenous H_2_S after 24 h and 48 h were measured. The data are means ± SE of three independent experiments. Different letters indicate significant differences (*P* < 0.05; Duncan’s multiple range test). FW, fresh weight

To investigate the total relationship between endogenous H_2_S content (endogenous H_2_S plus root absorbed exogenous H_2_S) and Cd stress in the roots of cucumber seedling, we measured the total endogenous H_2_S content at 24 and 48 h after the different treatments. As shown in Fig. 4, after 24 h of treatment, Cd stress, NaHS, and NaHS + Cd treatments could induce an increase in endogenous H_2_S content, which was significantly higher than that of the control (119.1, 50.7 and 170.6%, respectively), and the endogenous H_2_S content in NaHS + Cd treatment was significantly higher (24.2%) than that in the Cd treatment. The content of endogenous H_2_S in cucumber seedling roots decreased after 48 h compared with that of 24 h, while the endogenous H_2_S content under NaHS + Cd treatment was still 107.9, 28.8 and 78.5% higher than that of Con, Cd and NaHS treatments, respectively. These results indicated that the cucumber seedling roots absorbed exogenous H_2_S which contributed to the overall levels of endogenous H_2_S and helped to alleviate the Cd stress.

### H_2_S reduced the cd-induced accumulation of ROS, malondialdehyde (MDA) and the electrolyte leakage percentage (ELP)

**Fig. 5:**
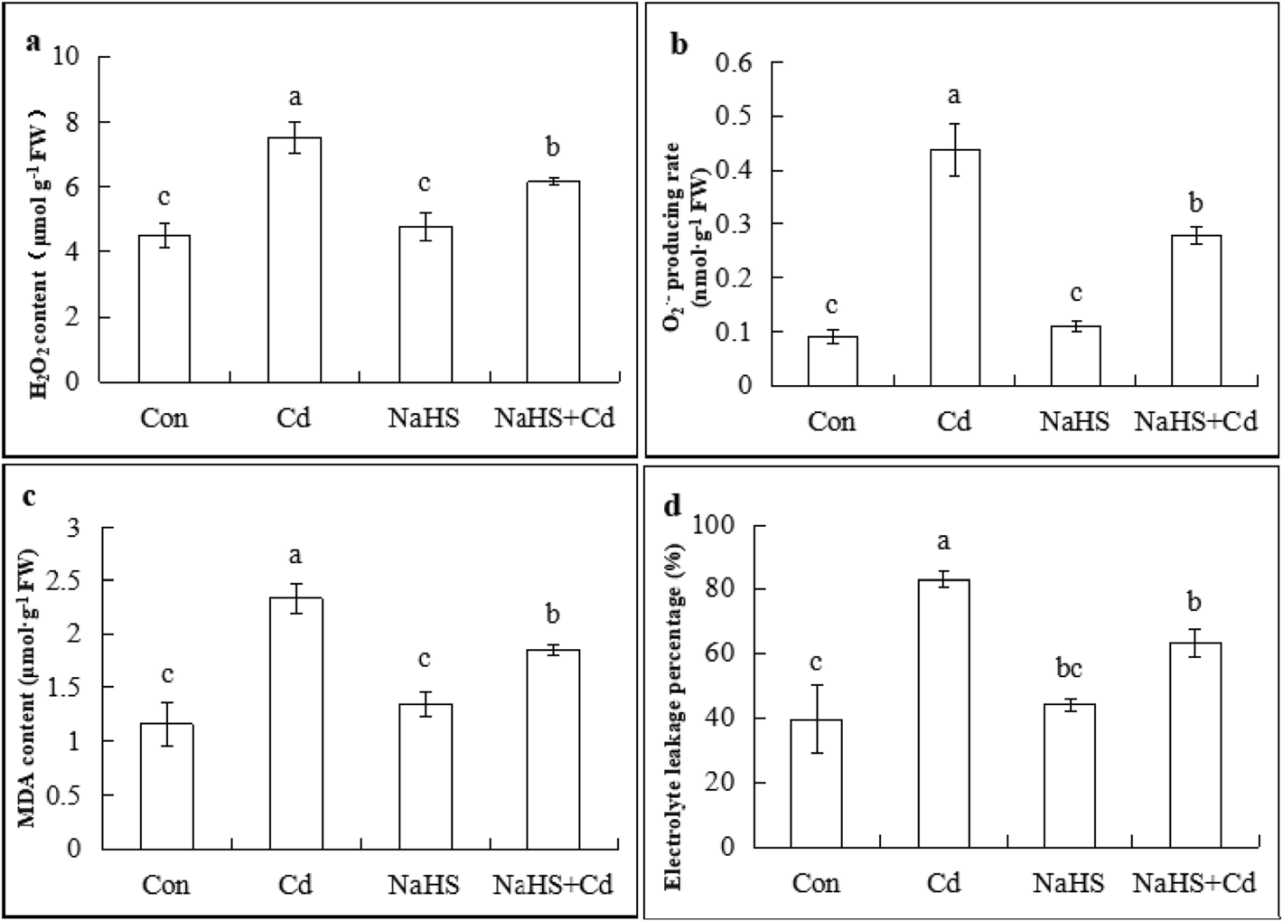
Effects of H_2_S on H_2_O_2_, O2·−, MDA and ELP under Cd stress in cucumber seedling roots. Cucumber seedlings pretreated with 100 μM NaHS were exposed to Cd stress for 48 h and analyzed for the content of H_2_O_2_ (**a**), O_2_^·−^ (**b**), MDA (**c**), and ELP (**d**). The data are means ± SE of three independent experiments. Different letters indicate a statistically significant difference (*P* < 0.05; Duncan’s multiple range test). FW, fresh weight

To determine whether H_2_S could regulate the level of ROS to decrease Cd-induced cell death, we measured H_2_O_2_ and O_2_^·−^ contents in the root tips of cucumber seedlings after 48 h of Cd stress (Fig. 5a, b). Exposure to 200 μM CdCl_2_ increased the production of H_2_O_2_ that was 66.7% higher than that of the control, whereas NaHS + Cd significantly decreased H_2_O_2_ content (17.8%). Under Cd treatment, the content of O_2_^·−^ was 3.89 times greater than that of the control. However, pretreatment with NaHS reduced the level of O_2_^·−^ by 36.4%. Therefore, pretreatment with NaHS effectively reduced ROS accumulation and thereby protected membrane integrity under Cd stress.

As shown in Fig. 5c, the highest value for MDA content was recorded under Cd stress, whereas the lowest values for MDA content were obtained with the control and NaHS alone. Moreover pretreatment with NaHS significantly reduced MDA content by 20.6% compared to that of the Cd stress treatment. Similarly, the NaHS + Cd treatment decreased ELP by 23.7% (Fig. 5d). The results showed that H_2_S inhibited lipid peroxidation of cell membrane to ensure the integrity of cell structure and improve the tolerance of the plants to Cd stress.

### H_2_S activated antioxidant enzymes to reduce oxidative damage

**Fig. 6:**
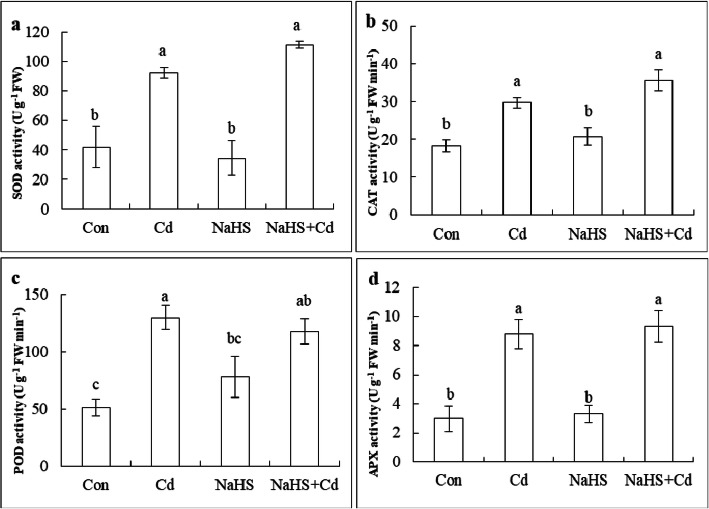
Effects of H_2_S on antioxidant enzyme activity under Cd stress in cucumber seedling roots. Cucumber seedlings pretreated with 100 μM NaHS were exposed to Cd stress for 48 h and analyzed the activity of SOD (**a**), CAT (**b**), POD (c), and APX (**d**). The data are means ± SE of three independent experiments. Different letters indicate a statistically significant difference (*P* < 0.05; Duncan’s multiple range test). FW, fresh weight

To explore the reduction of ROS accumulation resulting from H_2_S, we measured SOD, CAT, POD and APX activity in the root tips of cucumber seedlings after 48 h of Cd treatment. As shown in Fig. 6, under Cd stress, SOD, CAT, POD and APX activity markedly increased by 119.7%, 63.5, 130.3 and 194.9%, respectively, compared with that of the control, whereas the control and NaHS treatment alone did not exhibit a significant difference. Compared with the Cd treatment, NaHS + Cd improved SOD, CAT, POD and APX activity by 20.5, 20.0, 10.3 and 0.6% respectively. These results showed that the reduction of ROS accumulation by H_2_S depended on the activation of the antioxidant enzyme system.

### Effect of NaHS on DAPI staining and fluorescence quantitative analysis

**Fig. 7:**
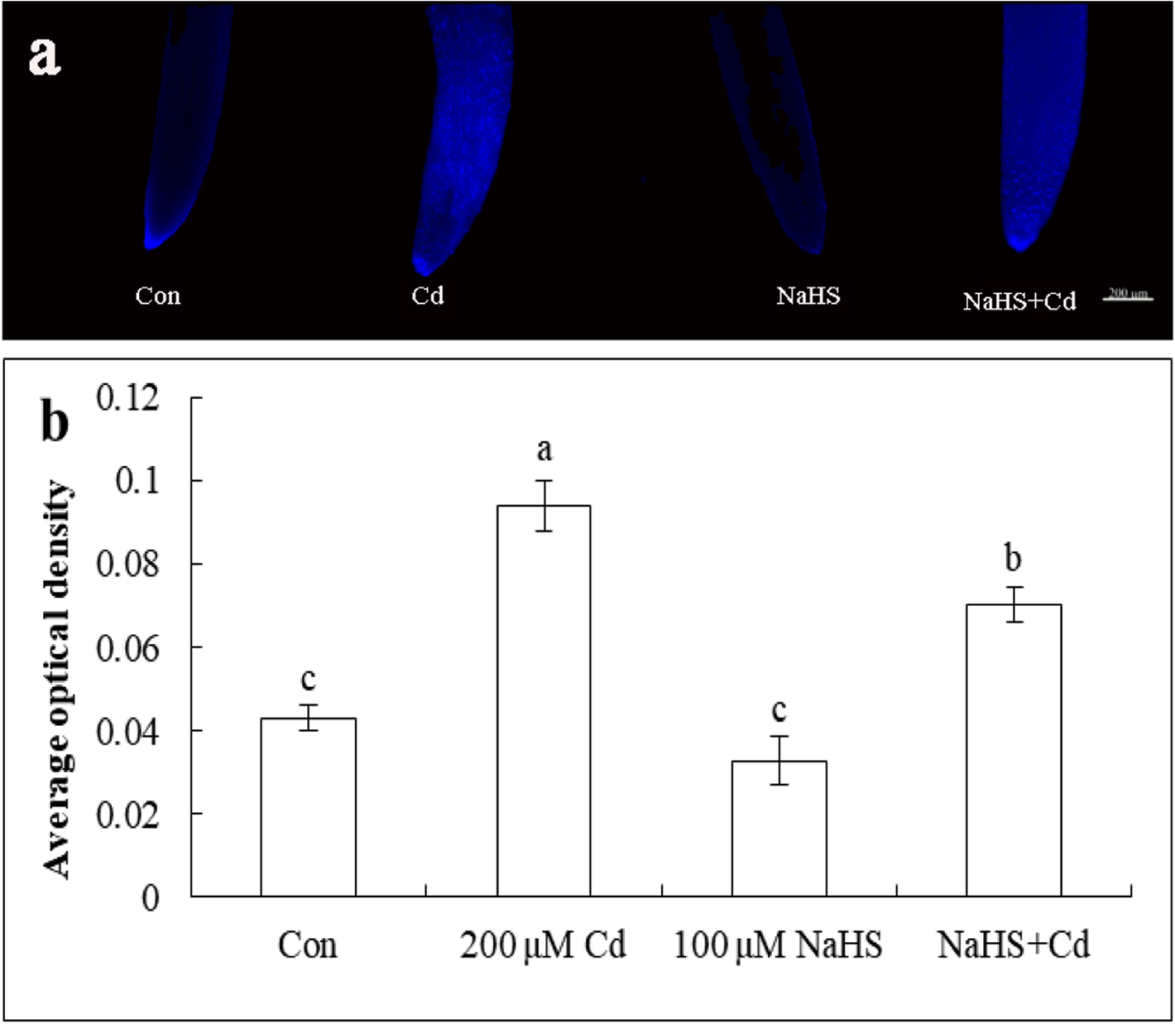
DAPI staining (**a**) and fluorescence quantitative analysis (**b**). Con: distilled water; Cd: 200 μM CdCl_2_; NaHS: 100 μM NaHS pretreatment for 24 h; NaHS + Cd: 100 μM NaHS pretreatment for 24 h + Cd treatment for 48 h. The data are means ± SE of three independent experiments. Different letters indicate significant differences (*P* < 0.05; Duncan’s multiple range test)

To investigate the effects of NaHS on cell death in root tips of cucumber seedlings, DAPI staining was used as a diagnostic marker for cell death. As shown in Fig. 7a, weak fluorescence was observed in the control and NaHS pretreatment. The root tips of cucumber seedlings under Cd stress showed strong fluorescence after 48 h; however, the NaHS + Cd treatment reduced the fluorescence. Quantitative fluorescence analysis also showed that the NaHS + Cd treatment could significantly reduce fluorescence by 25.2% compared with the Cd treatment (Fig. 7b), indicating that NaHS pretreatment reduced Cd-induced cell death in root tips as measured by DAPI staining.

### H_2_S inhibited Cyt c release from the mitochondria and caspase-3-like activity under cd stress

**Fig. 8:**
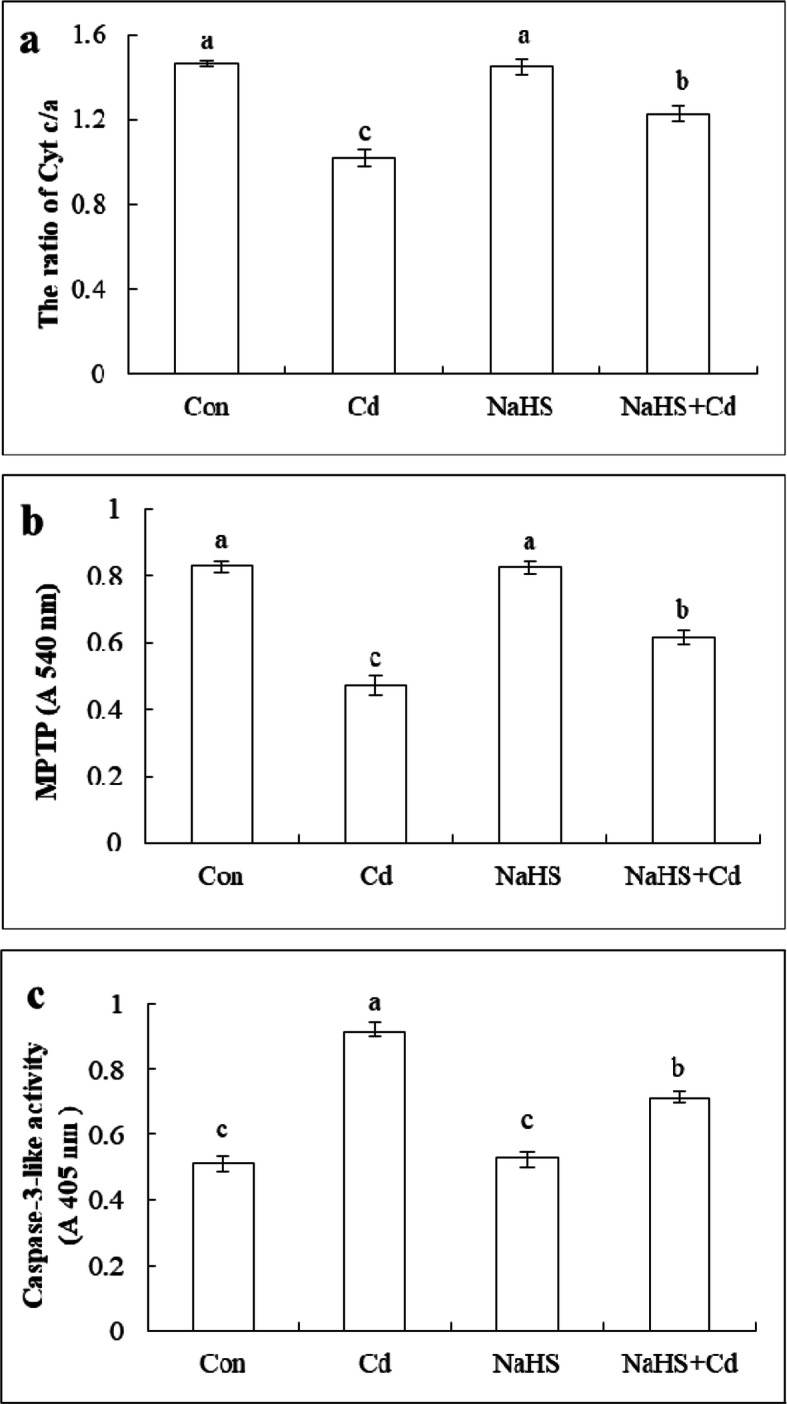
Effects of NaHS on mitochondrial Cyt c/a, MPTP and caspase-3-like activity of cucumber seedlings root tips under Cd stress. Con: distilled water; Cd: 200 μM Cd stress for 48 h; NaHS: pretreated with 100 μM NaHS for 24 h; NaHS + Cd; seedlings were pretreated with 100 μM NaHS for 24 h and then treated with 200 μM Cd for 48 h. The ratio of Cyt c/a (**a**), mitochondrial membrane absorbance (**b**) and caspase-3-like (**c**) were measured after 48 h in different treatments. The data are means ± SE of three independent experiments. Different letters indicate significant differences (*P* < 0.05; Duncan’s multiple range test)

To investigate whether H_2_S affected mitochondrial Cyt c release under Cd stress, the content of Cyt c/a and MPTP were measured. Cyt c was loosely bound to the phospholipids of the mitochondrial inner membrane, and could not freely pass through the outer mitochondrial membrane, whereas Cyt a was tightly bound to the inner mitochondrial membrane. Thus Cyt c/a could indicate the Cyt c content in the mitochondrial inner membrane. As shown in Fig. 8a, Cd treatment significantly reduced the ratio of Cyt c/a by 43.8% compared with that of control. The NaHS + Cd treatment significantly increased the Cyt c/a value by 22.6% compared with that of the Cd treatment, but there was no difference between the control and H_2_S pretreatment. As shown in Fig. 8b, Cd stress significantly reduced the mitochondrial membrane absorbance, whereas those of the control, and the NaHS and NaHS + Cd treatment were higher than that of Cd treatment alone by 75.3, 74.5 and 30.2% respectively.

Caspase-3 plays an important role in animal cell apoptosis and there have been similar studies in plants. To investigate whether H_2_S affected the caspase-3-like activity, enzyme activity was measured at 48 h after treatments. As shown in Fig. 8c, caspase-3-like activity increased significantly, and was 77.8% higher than that of the control. NaHS pretreatment for 24 h significantly reduced caspase-3-like activity, which was 22.1% lower than that of the Cd treatment.

Taken together, the results indicated that Cd stress could lead to Cyt c release into the cytoplasm, increase the degree of opening of MPTP, and activate caspase-3-like activity, whereas NaHS pretreatment could inhibit these effects and reduce the release of Cyt c from the mitochondria and caspase-3-like activity.

## Discussion

Some studies have shown that Cd interferes with plant metabolism and physiological processes, such as reducing root length [40, 41] and leaf area [42] and causing cell death [43]. Cd stress seriously affected the root development and fresh weight of Chinese cabbage [44]. In barley plants, high concentration of Cd for 9 h retarded growth and cell death was observed compared with low concentration of Cd [45]. In this experiment, Cd inhibited root elongation and reduced fresh weight of cucumber seedlings. With an increasing Cd concentration the inhibitory effect was significantly enhanced (Fig. 1). Some studies have shown that Cd stress can cause plant cell death. Leaf cell death of the submerged angiosperm *Ruppia maritima* was observed after 3 or 5 d exposure to Cd [46]. The 10 μM and 100 μM Cd treatments resulted in the cell death which appeared between 24 and 48 h and between 12 and 24 h in maize, respectively [47]. Our results revealed that the longer the duration of exposure to 200 μM Cd, the greater the number of cells that die (Fig. 2). Similarly, Zhang et al. [48], found that 5 mM Cd treatment led to cell death in Chinese cabbage roots.

H_2_S is the third physiologically gas signal molecule in both animals and plants with versatile functions and it plays a vital role in alleviating heavy metal stress. Under aluminum (Al) stress, barley seedlings root elongation was inhibited, whereas pretreatment with NaHS effectively alleviated the inhibition of root elongation induced by Al [49]. In *Solanum nigrum* L. seedlings, H_2_S regulated the distribution and absorption of zinc (Zn) in roots, thereby alleviating the stress caused by Zn on root development [50]. H_2_S is a common gas molecule occurring in response to heavy metal (HM) stress, and Cd is among the HMs that are very toxic and causes severe stress in plants [51]. In Fig. 3a, b, 100 μM NaHS pretreatment significantly reduced the inhibition of Cd stress on root length and fresh weight of cucumber seedlings. This was consistent with other reports that showed that H_2_S improved plant tolerance to Cd stress in plants, such as the foxtail millet [52], *Brassica napus* [53], and *Arabidopsis* [54]. Meanwhile we observe decreased cell death after the pretreatment of cucumber with 100 μM NaHS under Cd stress (Fig. 3c, d). Similarly, Cheng et.al reported that pretreatment with exogenous NaHS significantly alleviated hypoxia-induced root tip death in pea seedlings and enhanced the tolerance to hypoxic stress [55]. Furthermore, Zhang et.al also reported that NaHS pretreatment could reduce cell death in Chinese cabbage root and promoted root elongation under Cd stress [48]. Our results suggested that H_2_S protects cucumber roots from Cd-induced root cell death.

ROS are by-products of plant aerobic respiration, and their steady level depends on the interaction between ROS-producing and ROS-scavenging mechanisms. Excessive ROS can cause damage to plants, including membrane peroxidation, protein denaturation, enzyme inactivation and DNA damage, which can cause cell death [56]. In *Arabidopsis thaliana*, Cd can active the MPK3/ MPK6 signal pathway in a ROS dosage-dependent manner [57]. A high concentration of Cd increased H_2_O_2_ and O_2_^·−^ contents, which led to oxidative damage followed by root growth inhibition and cell death. Moreover endogenous H_2_S participated in the reduction of the ROS level through the up-regulation of *Br_UPB1s* in the root tips of *Brassica rapa* [58]. In this study, we also found that the content of H_2_O_2_, O_2_^·−^, MDA and ELP in root of cucumber seedlings increased significantly, and H_2_S could inhibit oxidative damage and membrane peroxidation by activating the antioxidant enzyme system after 48 h of Cd treatment (Figs. 5, 6). Kaya et.al also reported that H_2_S could improve the activities of antioxidant enzymes and reduced oxidative stress to alleviate the toxicity of Cd in wheat and strawberries [7, 59]. Metal salts such as Al, iron (Fe) and Cd can induce cell death in plants. When two genotypes of rice, Azucena (iron tolerant) and IR64 (iron sensitive) were exposed to Fe^2+^ (400 mM) stress, it induced cell death in the root tips of the IR64 cultivar [60]. Under 89 mM CdCl_2_ treatment, roots of 3-d-old yellow lupine (*Lupinus luteus* L.) seedlings suffered PCD after 24 h, which was observed by TUNEL-positive reaction [61]. Similarly, we also observed the occurrence of cell death by DAPI staining and fluorescence quantitative analysis (Fig. 7). ROS such as O_2_^·−^ and H_2_O_2_, induce cell death in plant and animal cells [62]. ROS level burst is the most important signal involved in Cd-induced cell death in plants [63, 64]. It was reported that Cd-induced cell death in suspension cells of *Arabidopsis thaliana* was accompanied by an increase in H_2_O_2_ content [65]. We found that NaHS pretreatment reduced the accumulation of ROS (Fig. 5) and inhibited the occurrence of cell death (Fig. 7**)** by increasing antioxidant enzyme activity (Fig. 6). Our results are consistent with those of Zhang et al. who reported that NaHS treatment delayed the cell death process in gibberellic acid- (GA-) treated aleurone layers, where it reduced ROS level and increased antioxidant enzymes activity [66]. The up-regulation of antioxidant enzymes related genes by H_2_S decreased the accumulation of H_2_O_2_, and thus inhabited Cd-induced DNA fragmentation and chromatin condensation [67].

Cyt c is located in the inner membrane of the mitochondria, and involves the electron transfer of the respiratory chain in normal cells, but it cannot penetrate the outer membrane of the mitochondria. Different apoptotic inducible factors can induce the release of Cyt c and activate cell death, such as heat shock [37], H_2_O_2_ [68] and Al stress [69]. Our experiments also confirmed that Cd caused Cyt c to detach from mitochondria into the cytoplasm and the opening of MPTP (Fig. 8a, b). Furthermore, pretreatment with 100 μM NaHS weakened the negative effect of Cd stress that promoted the release of Cyt c and the opening of MPTP. This was consistent with earlier reports that indicated that H_2_S inhibits cell apoptosis by inhibiting Cyt c release, such as in SH-SY5Y cells [70], RGC-5 cells [71] and rat cells [72]. Cysteine protease is a kind of biological and cytokine maturation and apoptosis protease is a class of the cysteine protease family. The Cysteine protease family is the main regulator of the cell death mechanism. When caspase proteasome is stimulated and activated, the cell death process is initiated, leading to the execution of cell death regulation [73]. Plant cysteine protease is similar to caspase protease in the apoptotic process in animal cells. It also participated in the regulation of cell death in plant cells. Poly (ADP-ribose) polymerase (PARP) participated in plant cell death induced by H_2_O_2_, and the degradation of plant PARP depended on the release of Cyt c into the cytoplasm, which could be inhibited by specific caspase-3 inhibitors [74]. Ye et al. reported that 100 μM Cd led to an increase of caspase-3-like activity in *Arabidopsis* suspension cells [75]. Our results also indicated that a Cd-induced increase in the caspase-3-like activity in cucumber seedling root tips (Fig. 8c). Moreover, exogenous NaHS pretreatment reduced the increase of caspase-3-like activity induced by Cd stress. Similarly, H_2_S improved mitochondrial dysfunction and suppressed the ROS-mediated caspase-3 pathway in cortical neurons [76]. H_2_S could inhibit apoptosis induced by high-glucose toxicity in rat peritoneal mesothelial cells by decreasing caspase-3 activity [77].

In summary, our data revealed that H_2_S inhibits Cd-induced cell death in root tips of cucumber seedlings. The application of Cd inhibited root elongation growth, caused cell death and was accompanied by a release of mitochondrial Cyt c, the opening of MPTP and increase in caspase-3-like activity. Pretreatment with exogenous H_2_S donor, NaHS, inhibited the occurrence of Cd-induced cell death by reducing ROS accumulation, Cyt c release and caspase-3-like activity. This study suggested that the possible mechanism of H_2_S protection of cucumber seedling roots from cell death under Cd stress, although future experiments are needed to determine how this protective effect could be applied in cucumber production.

## Methods

### Plant material and treatments

Cucumber (*Cucumis sativus* ‘Xinchun 4’) seeds were obtained from Gansu Academy of Agricultural Sciences, Lanzhou, China. In experiment 1, the seeds were placed in Petri dishes lined with filter papers and the seeds were germinated in darkness at 28 °C for 48 h. Then, the 2-d-old seedlings were treated with different concentrations of cadmium chloride (50, 100, 200 and 300 μM CdCl_2_) and transferred to an illuminating incubation climate box (25 ± 1 °C, 12 h photoperiod, photo- synthetically active radiation = 200 μmol m^− 2^ s^− 1^) for 48 h. In experiment 2, seeds germinated for 24 h were pretreated with different concentrations (1, 10, 100 and 200 μM) of sodium hydrosulfide (NaHS, a H_2_S donor) solution for 24 h, and then the seedlings were exposed to 200 μM CdCl_2_ for 48 h. Then the root length and fresh weight of cucumber seedling were measured.

### Detection of cell death

Evans blue staining has been widely used as an indicator of dead cells. According to the method of Zhang [48], the roots (2 cm long) of seedlings which were treated for 48 h were stained with 0.25% (w/v) Evans blue for 15 min and washed with water three times. The roots were observed under a microscope and pictures were taken (Revolve RVL-100-G, ECHO, USA). After staining, the roots were homogenized with 1 mL 80% ethanol and incubated 15 min at 50 °C, then centrifuged at 10, 000 *g* for 10 min, then the absorbance was measured at 600 nm.

### Determination of endogenous H_2_S content

According to the method of Fang [78], 0.2 g of a root tip-sample was added to 5 mL of 50 mM phosphate buffer solution (0.2 M ascorbic acid (AsA), 0.1 M EDTA and 0.5 mL 1 M HCl, pH 6.8), which was added to the homogenate. The released H_2_S was collected by 1% (w/v) zinc acetate. After 30 min, 0.3 mL 5 mM dimethyl-p-phenylenediamine dissolved in 3.5 mM H_2_SO_4_ was added to the mixture and then 0.3 mL of 50 mM ferric ammonium sulfate was added. After 15 min of reaction, the value of absorption at 667 nm was detected.

### Hydrogen peroxide (H_2_O_2_) and superoxide anion radical (O_2_^·−^) analysis

To determine H_2_O_2_ content after Cd stress for 48 h in root, we weighed 0.2 g of a root tip-sample and ground it with pre-cooled acetone. This was then transferred to centrifugal tube for 3000 rpm centrifugation for 10 min at 4 °C. Extract (1 mL) was added to 0.1 mL 5% titanium sulfate and 0.2 mL concentrated ammonia water; precipitation was centrifuged at 3, 000 rpm for 10 min at 4 °C, and then the precipitate was washed for 3–5 times with acetone. Finally, 2 mol L^− 1^ euphoric acid was added to the precipitate and colorimetric analysis was carried out at 415 nm [79].

To determine the O_2_^·−^ content, root samples (0.2 g) were homogenized with 1 mL of phosphate buffer (pH 7.8) and centrifuged at 12, 000 rpm for 15 min at 4 °C. Hydroxylamine hydrochloride (1 mL) was added to the supernatant to react for 1 h and then 1 mL of p-aminobenzene sulfonic acid and 1 mL of α-naphthylamine were added to the mixture. The solution was kept at 25 °C for 20 min. The value of absorption at 530 nm was detected [49].

### Measure of malondialdehyde and ELP

A 0.2 g root sample was added to the thiobarbituric acid reaction and the reaction solution was immersed in a water bath at 95 °C for 20 min, and then cooled to room temperature. Finally, the absorbance values were measured at 450, 532, and 600 nm, respectively [80].

A 0.2 g root sample was added to 10 mL distilled water and incubated at 25 °C for 2 h. Then, the solution was read by electrical conductivity (EC1). Finally, samples were treated in the water-bath at 95 °C for 30 min and then read for EC2, where ELP = EC1/EC2 × 100% [81].

### Antioxidant enzyme activity assay

Antioxidant enzyme activities were measured by the methods described by Bu et al. [82]. Roots (2 cm long) were added to 1.5 mL of 50 mM PBS buffer (1 mM EDTA and 1% polyvinylpyrrolidone) and homogenized. The homogenate was centrifuged at 10, 000 *g* for 10 min at 4 °C and the extract was used to detect the activity of antioxidant enzymes.

Superoxide dismutase (SOD) activity was measured at 560 nm, based on inhibiting the photochemical reduction of nitroblue tetrazolium (NBT). Peroxidase (POD) activity was assayed by minor modifications; enzyme extract (0.1 mL) was added to 2.6 mL guaiacol (0.3% in 50 mM phosphate buffer, pH 6.5) and 0.3 mL 0.6% H_2_O_2_, then the change in absorbance was measured at 470 nm for 2 min. Catalase (CAT) was measured at 290 nm for 3 min, using the 2% H_2_O_2_, 50 μL enzyme solution and 50 mM PBS buffer. Ascorbate peroxidase (APX) was determined at 290 nm for 3 min, using 50 mM PBS buffer, 15 mM ascorbate, 50 μL of enzyme extract and 30 mM H_2_O_2_.

### 4, 6-Diamidino-2-phenylindole (DAPI) staining

Measurement of DAPI staining following a previously described method [83] with some modifications. After fixing a 2 cm long root with 4% glutaraldehyde solution for 24 h, it was washed with distilled water three times. The roots were immersed in 1 μg mL^− 1^ (w/v) DAPI for 10 min at room temperature, followed by washing several times in distilled water. DAPI stain image was viewed with a fluorescence microscope (Revolve RVL-100-G, ECHO, USA). The fluorescence intensity was measured via ImageJ software.

### Detection of the ratio of Cyt c/a and mitochondrial permeability transition pore (MPTP) in mitochondria

As described in a previous study [84], mitochondria were isolated from root tips of 4 d-old cucumber seedlings using a kit (Bestbio, BB-3611-1, China) following the manufacturer’s instructions. The suspended mitochondria were added with 0.2% BSA to reach final concentration of 0.5 mg mL^− 1^, the absorbance of the suspension was measured with an ultraviolet spectrophotometer at 550 nm and 630 nm. The ratio of Cyt c/a = A550 / A630.

The isolated mitochondria were suspended with buffer II (5 mM sodium succinate, 70 mM sucrose, 5 mM HEPES, 220 mM mannitol, pH 7.2), the concentration of the adjusted protein was 0.3 mg/mL and then incubated at 20 °C for 2 min. The absorbance was recorded at 540 nm [69].

### The activity of caspase-3-like

The caspase-3-like assay kit (Solarbio, BC3830) was used according to the manufacturer’s instructions. To measure the activity of caspase-3-like, assays were performed in 96-well microtitrer plates by incubating 35 μL extraction solution + 65 μL reaction buffers [5 μL caspase-3 substrate (DEVD-*p*NA), 2 mM]. Lysates were incubated at 37 °C for 4 h. The mixture was measured with an ELISA reader (CMax Plus, Molecular Devices, USA) at 405 nm.

### Statistical analysis

All the values in this study were repeated three times, and the results shown are the mean ± standard error (SE) of three independent experiments. Data analysis was used for Duncan’s multiple range test (*P* < 0.05) using SPSS 19.0 software (IBM SPSS, Chicago, USA).

## Data Availability

The datasets generated during the current study are available from the first author on reasonable request.

## Abbreviations

H_2_S: Hydrogen sulfide
PCD: Programmed cell death
ROS: Reactive oxygen species
H_2_O_2_: Hydrogen peroxide
O_2_^·−^: Superoxide anion radical
MDA: Malondialdehyde
ELP: Electrolyte leakage percentage
SOD: Superoxide dismutase
POD: Peroxidase
CAT: Catalase
APX: Ascorbate peroxidase
Cyt c: Cytochrome c
MPTP: Mitochondrial permeability transitions pore
GA: Gibberellic acid
DAPI: 4, 6-Diamidino-2-phenylindole
